# Effects of Microplastics Exposure on the *Acropora* sp. Antioxidant, Immunization and Energy Metabolism Enzyme Activities

**DOI:** 10.3389/fmicb.2021.666100

**Published:** 2021-06-04

**Authors:** Baohua Xiao, Dongdong Li, Baolin Liao, Huina Zheng, Xiaodong Yang, Yongqi Xie, Ziqiang Xie, Chengyong Li

**Affiliations:** ^1^Shenzhen Institute of Guangdong Ocean University, Shenzhen, China; ^2^Southern Marine Science and Engineering Guangdong Laboratory, School of Chemistry and Environment, Guangdong Ocean University, Zhanjiang, China

**Keywords:** microplastics, *Acropora* sp., endosymbiont, enzyme, biochemical evaluation

## Abstract

Microplastic pollution in marine environments has increased rapidly in recent years, with negative influences on the health of marine organisms. Scleractinian coral, one of the most important species in the coral ecosystems, is highly sensitive to microplastic. However, whether microplastic causes physiological disruption of the coral, *via* oxidative stress, immunity, and energy metabolism, is unclear. In the present study, the physiological responses of the coral *Acropora* sp. were determined after exposure to polyethylene terephthalate (PET), polyamide 66 (PA66), and polyethylene (PE) microplastic for 96 h. The results showed that there were approximately 4–22 items/nubbin on the surface of the coral skeleton and 2–10 items/nubbin on the inside of the skeleton in the MPs exposure groups. The density of endosymbiont decreased (1.12 × 10^5^–1.24 × 10^5^ cell/cm^2^) in MPs exposure groups compared with the control group. Meanwhile, the chlorophyll content was reduced (0.11–0.76 μg/cm^2^) after MPs exposure. Further analysis revealed that the antioxidant enzymes in coral tissues were up-regulated (Total antioxidant capacity T-AOC 2.35 × 10^–3^–1.05 × 10^–2^ mmol/mg prot, Total superoxide dismutase T-SOD 3.71–28.67 U/mg prot, glutathione GSH 10.21–10.51 U/mg prot). The alkaline phosphatase (AKP) was inhibited (1.44–4.29 U/mg prot), while nitric oxide (NO) increased (0.69–2.26 μmol/g prot) for cell signal. Moreover, lactate dehydrogenase (LDH) was down-regulated in the whole experiment period (0.19–0.22 U/mg prot), and Glucose-6-phosphate dehydrogenase (G6PDH) for cell the phosphate pentoses pathway was also reduced (0.01–0.04 U/mg port). Results showed that the endosymbiont was released and chlorophyll was decreased. In addition, a disruption could occur under MPs exposure, which was related to anti-oxidant, immune, and energy metabolism.

## Graphical Abstract

The experiment flow chart of anti-inflammatory and analgesic effects of *P. orientale* extracts.

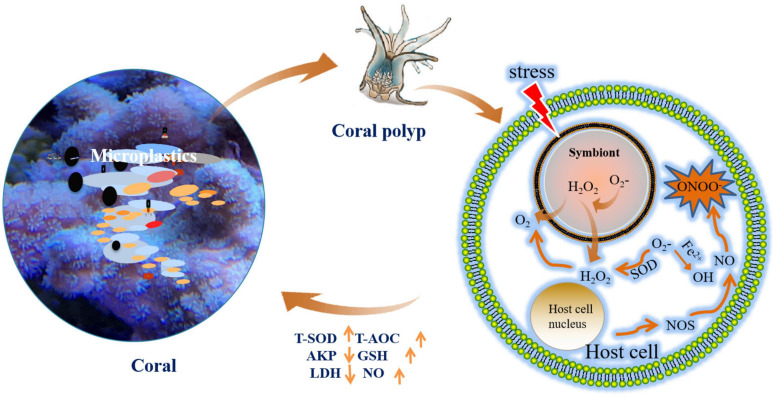

## Introduction

*Acropora* sp., a species of scleractinian coral, is a complex symbiosis constituting with scleractinian host, photosynthetic symbionts, and various microbial communities (Besseling et al., 2014; Tian and Niu, 2017; Yu et al., 2020a,b). Corals supply protection and inorganic salt for the endosymbiont, and in return, the endosymbiont provides its host with organic nutrients (Cook and D’ Elia, 1987). Although corals can obtain energy from symbiotic endosymbiont, they need to ingest extra exogenous food to satisfy their nutrition (Allen et al., 2017; Tian and Niu, 2017). However, the intricate relationship between coral and endosymbiont symbiosis is threatened by environmental changes such as global climate change and aquatic environment pollution (Hughes et al., 2017; Saliu et al., 2019; Yu et al., 2020a,b). Global coral reefs are suffering from continual and serious degradation in recent years (Veron, 1992; Zhao et al., 2013, 2016; Higuchi et al., 2015a,b; Hughes et al., 2017).

An estimated approximately 8–12 million tons of various plastic waste are transferred into the ocean in multiple ways each year (Carpenter et al., 1972; Hidalgo-Ruz et al., 2012). Previous reports show that plastic waste accounted for 70–90% of marine waste (Lusher, 2015; Walker, 2018). Lamb assessed the influence of plastic waste on reef-building corals in the Asia-Pacific region, and they found that plastic waste increased the risk of diseases in corals from 4 to 89% (Lamb et al., 2018). Furthermore, in the marine environment plastic waste can develop into small fragments through biodegradation, thermal degradation, hydrolysis, and photodegradation (Hidalgo-Ruz et al., 2012; Walker, 2018). Microplastics (MPs) are described as plastic pieces smaller than five millimeters, and they are more difficult to manage than other pollutants due to this small size and global distribution (Antao Barboza and Garcia Gimenez, 2015; Ali Chamas et al., 2020). MPs can be ingested by a wide range of marine organisms, and they bring negative effects, including gastrointestinal obstruction, inflammation, tissue damage, and growth restriction (Sun et al., 2017; Wang et al., 2020). Due to the stable chemical composition, MPs are difficult for marine organisms to digest. Hence, MPs accumulate continuously in marine organisms (Deudero and Alomar, 2015; Sun et al., 2017).

MPs are mainly discharged from terrestrial environments into the sea, meaning coastal ecosystems such as coral reefs are especially at risk (Hermabessiere et al., 2017; Huang et al., 2019). The threat of numerous MPs to coral reefs has attracted extensive attention (Kirstein et al., 2016; Reichert et al., 2018). Reichert found that MPs could attach to the tentacles or skeleton surface of *Pocillopora damicornis*, and the coral was subject to germ infection, bleaching, and even tissue necrosis (Reichert et al., 2018). Additionally, Hankins evaluated the effects of MPs on *Montastraea cavernosa* and *Orbicella faveolat*. They found that the corals captured MPs actively although they could recognize and repel indigestible substances (Hankins et al., 2018). Interestingly, coral tends to ingest MPs because it is driven by chemoreception (Allen et al., 2017). Furthermore, a previous report suggested that excessive ingestion of MPs could induce the scleractinian coral *P. damicornis* to produce oxidative stress, which can lead to a decrease in the expression of stress-related protein and activate the MAPK/Nrf2 pathway (Jeong et al., 2016). MPs not only cause irreversible damage to coral hosts but also seriously threaten the survival of symbiotic algae. Su found that MPs can inhibit the growth of endosymbiotic algae by affecting its apoptosis and metabolism (Su et al., 2020). MPs negatively affect the photosynthesis activity of coral endosymbiontic microorganisms through reducing chlorophyll content and photochemical efficiency (Mao et al., 2018; Wu et al., 2019). Many studies to date have found that MPs could accumulate in an organism and cause endocrine disruption (Chapron et al., 2018; Syakti et al., 2019). It has been revealed that the reactive oxygen species (ROS) and antioxidant enzymes (SOD, CAT, and GSH) of coral are up-regulated after exposure to MPs (Chen et al., 2017; Tang et al., 2018; Dias et al., 2019). Oxidative damage could be caused by excessive ROS after exposure to MPs (Paul-Pont et al., 2016; Chen et al., 2017). Although exposure and ingestion of MPs have been reported in many coral reefs, there is still a shortage of research on the adverse effects of MPs on coral *Acropora* sp.

The present study aimed to provide detailed information about the physical and toxicity effects of MPs on corals. The scleractinian coral *Acropora* sp. was chosen to evaluate the effect of MPs. The biochemical level stress response of *Acropora* sp. was determined by the antioxidant enzyme (T-SOD, T-AOC, and GSH), immunocompetence [alkaline phosphatase (AKP), nitric oxide (NO)], phosphate pentoses pathway [lactate dehydrogenase (LDH), Glucose-6-phosphate dehydrogenase (G6PDH)]. This is the first study to assess the physiological responses of scleractinian coral exposed to MPs. It examines the activity of enzymes involving antioxidant capacity, immune response, and energy metabolism in different stages. The results provide new insights into the response of corals and stress reactions caused by different kinds of MPs.

## Materials and Methods

### Materials

Polyethylene terephthalate microplastics (PET), Polyethylene microplastics (PE), Nylon 66 microplastics (PA66) were purchased from Saierqun, Shanghai, China. The microscope images and size distributions of MPs are shown in Supplementary Figure 1. The BCA protein assay kit was offered by the Beyotime Institute of Biotechnology (Shanghai, China). We 4% paraformaldehyde, acetic acid, 0.9% saline were purchased from Sigma-Aldrich (St Louis, MO, United States). Assay kits for measuring the levels of LDH, G6DPH, GSH, AKP, NO, T-SOD, and T-AOC were purchased from Nanjing Jiancheng Bioengineering Institute (A020, A027, A006, A059, A012, A001, and A015, Nanjing, China).

### Experimental Design

#### Collection and Treatment of Corals

Corals of the genus *Acropora* were used in this investigation. *Acropora* sp. is very sensitive to changes in the anthropogenic ecosystem (Mendrik et al., 2020). *Acropora* sp. was collected from the surrounding waters of Shenzhen Nanao Island (22°33′50.78″−22°40′38.18″N, 114°30′35.62″−114°33′26.90″E, 18–25°C, depth 5–8 m) (Supplementary Figure 2), according to the statistical data of Meteorological Bureau of Shenzhen Municipality (1980.01-2018.12). The annual average temperature is 21.5°C, the annual average sunshine is 2,325.3 h, and the annual average rainfall is 1,348.4 mm. There are 6 months in a year when the total solar radiation is above 400 MJ/m^2^. Five corals were collected from this location and quickly put into the holding tank. The oxygen pump was used to supply oxygen to corals.

In the laboratory, the whole origin coral polyps were transferred to open flow system glass tanks (160 cm × 50 cm × 75 cm) at ambient conditions, glued on ceramic plates by cyanoacrylate. The coral’s acclimation modular system was conducted according to the previous description (Rocha et al., 2015). They were acclimated to the experimental conditions for 30 days. Subsequently, 2–5 cm long fragments were cut from the origin colonies and they were attached to the ceramic matrix bases with two-component glue. The branches in the colonies were split as nubbins, and 108 nubbins were thus generated in total. In addition, there were regular shape and single branch experimental corals with intact polyps on each nubbin. All nubbins were distributed equally in 15 L acrylic laboratory tanks filled with seawater. Corals were housed in a controlled tank with a temperature of 24 ± 1°C and a salinity of 35.0 ± 0.2 ppt. The whole coral nubbins were illuminated with blue-white fluorescent bulbs (Chihiros LED lighting system 21 W, A351M,^1^) at a light 70 ± 10 μmol quanta m^–2^ s^–1^ in a 10 h/14 h light-dark cycle for 30 days to adapt to the experimental environment.

#### Exposure of Microplastics

In the experiments, MPs (PET, PE, and PA66) were treated for *Acropora* sp. to optionally ingest. Prior to the experiment, all MPs were confirmed by Raman Spectrometer (RS, SR-510 Pro, Ocean optics Asia, 785 nm laser, Raman shift 50–3,500 cm^–1^).

In detail, seawater containing MPs was prepared by adding 250 mg MPs to a 100 mL beaker. Then 50 mL seawater was added into the beaker, and it was shaken well. The solution in the beaker was ultrasonic for 5 min (200 W). Finally, the solution in the beaker was mixed with 5 L of seawater, and constant stirring prevented MPs from depositing. The final concentration of MPs was 50 mg/L (9.0 × 10^10^ particles/L), which is similar to previous reports (Tang et al., 2018; Chantal et al., 2020). The control groups of corals nubbins were maintained in fresh seawater (three tanks). While the experiment groups were carried out in the PET group (three tanks), PA66 group (three tanks), and PE group (three tanks), which were each placed in seawater-containing MPs. Continuous gentle aeration was used to prevent the accumulation of MPs. There were 12 tanks with a capacity of 15 L in the present study. The temperature was controlled at 24–25°C by air conditioning. The seawater in all tanks was replaced once every 24 h with freshly filtered seawater from the coral culture system to ensure a suitable aquaculture water environment, and new MPs were also added at the same time.

### Separation of Microplastics

To obtain the concentration of MPs attached to the coral surface, a test was conducted based on Allen’s work (Allen et al., 2017). Briefly, the nubbins were placed in a glass beaker and then immersed in filtered seawater. The nubbins were sonicated (200 W) for 10 min to strip off MPs attached to the surface. The glass beaker was then placed at ambient temperature to settle for 1 day. After 24 h, all the solution was prudently decanted and filtered with 0.8 μm pore size glass fiber membranes (Beyotime Biotechnology, FF338).

To obtain the ingested MPs in corals, the coral tissues after sonic processing were immersed in 30% formic acid solution for 6 h, and then they were placed in excess KOH solution (ω = 10%). All the solution was collected. Filtered seawater was used to rinse undissolved corals to get remnant MPs and the solution was collected. The collected solution was blended and filtered through a glass fiber membrane to obtain MPs. To collect all MPs, the membrane was treated the same way, and then MPs were dried completely at room temperature. Finally, MPs were observed *via* a microscope. The number of MPs represented per coral nubbin (unit: items/nubbin). Because coral is a colony animal, the unit of items/nubbin was used to reflect the number of MPs in the coral during the analysis procedure.

### Measurement of Endosymbiont Density

The density of endosymbiont from corals was measured based on previous studies by Hedouin et al. (2016) and Higuchi et al. (2015a, b). The coral tissues were homogenized (60 Hz, 3 min, 4°C) in 5 ml of filtered seawater. Subsequently, the collected homogenates were mixed with 2 mL 4% paraformaldehyde and stored at 4°C for 30 min. 2 mL homogenate was resuspended with filtered seawater to count the number of endosymbiont per unit area by a hemocytometer (QIUJING, China). The coral nubbins surface area was measured according to the aluminum foil method (Johannes et al., 1970). Finally, the density of endosymbiont was expressed as the number of symbiont per unit area of the coral nubbins.

### Measurement of Chlorophyll

Chlorophyll from symbiotic algae after MPs exposure was analyzed as outlined in previous research by Stimson and Kinzie (1991). 2 mL homogenate was centrifuged at 2,500 rpm for 15 min under 4°C, and then the gathered symbiotic algae was centrifuged at 15,000 rpm for 30 s under 4°C. Subsequently, the centrifuged homogenate was extracted with 2 mL of 100% acetone for 24 h at 4°C. The absorbance of the extract was measured at wavelengths of 634, 647, 664, and 750 nm (Thermo NanoDrop 2000), respectively. The chlorophyll content was obtained according to the equations of Porra and Jeffrey (Jeffrey and Humphrey, 1975; Porra et al., 1989). The weight of chlorophyll was described as the chlorophyll content per unit area of coral nubbins (μg/cm^2^).

### Biochemical Evaluation of Coral Tissue

To get tissue homogenates, the coral tissue was moved into a 5 mL tube after weighing accurately, and it was added to nine times the volume of filtered seawater according to the ratio of m(g)/V(mL). The tissue was mechanically homogenized under ice bath conditions for making 10% homogenate that used an Automatic Sample Rapid Grinding Instrument (JingXin, Shanghai, China). The homogenate was centrifuged for 15 min at 5,500 rpm. Finally, the supernatant was transferred to a new tube, and then it was diluted with filtered seawater. After the total enzyme activities were obtained, the concentration of total protein in the supernatant was quantified using the BCA method (Zhou et al., 2018). Biochemical parameters were analyzed after the diluted supernatant was transferred to a new tube. The commercial kits were used to detect the activities of T-SOD, T-AOC, AKP, GSH, G6PDH, LDH, and the content of NO.

### Histology Observation

After exposure, the coral nubbins were fixed in 4% formalin-seawater for more than 24 h, then rinsed with filtered seawater and preserved in 70% ethanol and 30% seawater (V/V). The coral samples were immersed in ethylene diamine tetraacetic acid (EDTA) decalcifying solution (pH 7.2) for 2 weeks, and the solution was replaced at 48 h. The tissue was paraffin-embedded and sectioned (6 μm) in a Jinhua automatic tissue processor (Zhejiang, KEDD-BM-6L). At least five slices were made from each sample (each slide was from a different area and depth in the tissue). Comparisons were made among slides from the same area (tissue depth or polyp area). Chlorophyll distribution was observed and photographed *via* a fluorescence microscope (Japan, Nikon Type 108, blue light excitation).

The green fluorescence was obtained by the camera system (NIS-Elements).

### Statistical Analyses

The values were evaluated by one-way ANOVA and multiple analyses of variation using Statistical Analysis Software (SPSS 17.0 IBM, Armonk, NY, United States). Data were expressed as mean ± standard deviation (SD). In all cases, *p* < 0.05 was considered as a statistically significant difference. The asterisk (^∗^) expressed as the significant difference between the control and MPs treatment groups. Letter of a, b and c represented the differences of PET vs. PA66, PET vs. PE, and PA66 vs. PE, respectively.

## Results

### Microplastics in Corals

**FIGURE 1:**
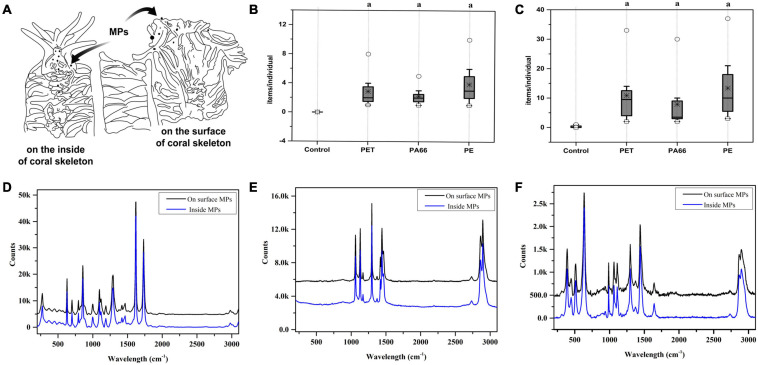
Plastic ingestion and Raman spectroscopy identification of microplastics structures in corals. The diagram of coral skeleton **(A)**. The MPs content on the inside of the skeleton **(B)** and on the surface of the coral skeleton **(C)** respectively. ○, *, and □ represent the maximum value, median value, and minimum value. Values are means ± SD, *N* > 3. Parts a represent significantly different groups (Wilcoxon test, *p* < 0.05). MPs identified by Raman spectroscopy on the surface of the skeleton and inside, and the results are presented respectively. **(D)** PET, **(E)** PE, and **(F)** PA66.

Figure 1A is a sketch that displays the distribution of MPs on the inside and outside of corals. Granular aggregation MPs could be found inside of corals (2–10 items/nubbins, Figure 1B) and on the surface (4–22 items/nubbins, Figure1C). The contents of the three kinds of MPs (PET, PA66, and PE) on the surface were higher than the ones inside (Figures 1B,C). Raman spectroscopy was used to distinguish the constituent (Figures 1D–F) of MPs. The results showed that the granular aggregations were PET (D), PE (E), and PA66 (F).

### The Impact of Microplastics Exposure on Endosymbiont and Chlorophyll

**FIGURE 2:**
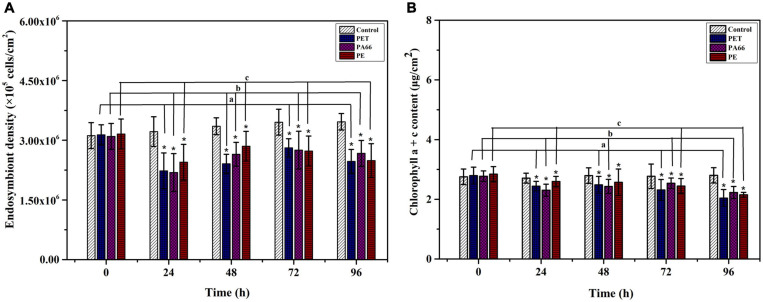
Effects of microplastics exposure on the density and chlorophyll content of endosymbiont in corals. Density variation of the endosymbiont in *Acropora pruinosa* after different MPs exposure **(A)**. Content variation of the chlorophyll a + c **(B)**. Histogram data represent means and error bars represent standard deviations (*N* > 3). Asterisk (*) represent a significant difference from normal (*p < 0.05).

The density of endosymbiont in corals is stable in the control group (*p* > 0.05) (Figure 2A). However, after exposing for 96 h, their densities were lower (*p* < 0.05) in all MPs exposure groups (PET 2.47 × 10^5^ cell/cm^2^, PA66 2.67 × 10^5^ cell/cm^2^, and PE 2.49 × 10^5^ cell/cm^2^) compared with the control group (3.46 × 10^5^ cell/cm^2^). The density of endosymbiont was the lowest after 24 h of MPs treatment, indicating that endosymbiont was sensitive to MPs toxicological reaction. The chlorophyll content of corals is shown in Figure 2B. The chlorophyll a + c content was stable in the control group (*p* > 0.05). Compared with the control group, it reduced to the lowest value (*p* < 0.05) at 96 h in the presence of PET, PA66, and PE. In general, the chlorophyll content was reduced in all MPs treatment groups at 96 h.

### Fluorescence Analysis of the Chlorophyll in Corals

**FIGURE 3:**
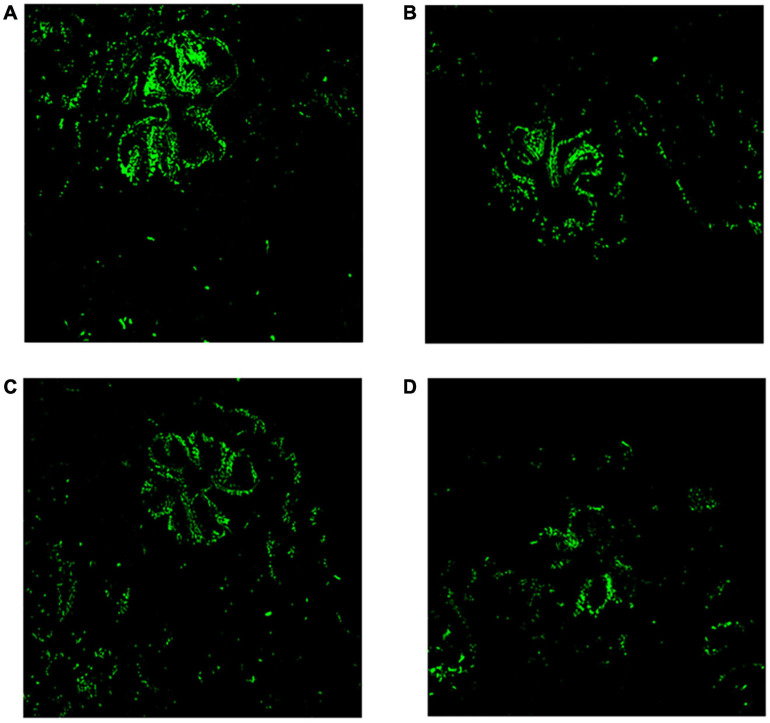
Microcosmic analysis of corals after MPs exposure for 96 h. Fluorescence analysis of chlorophyll in coral tissue (20×). **(A)** Control, **(B)** PET, **(C)** PA66, and **(D)** PE.

The chlorophyll green fluorescence of the control group was more than those corals with MPs treatment groups (see in Figure 3). Short-term high concentration of MPs exposure caused damage and disturbed the symbiosis with endosymbiont.

### Effect of Microplastics Exposure on Biochemical Indices of Coral

#### The Effect of MPs on Anti-Oxidative Capacity

**FIGURE 4:**
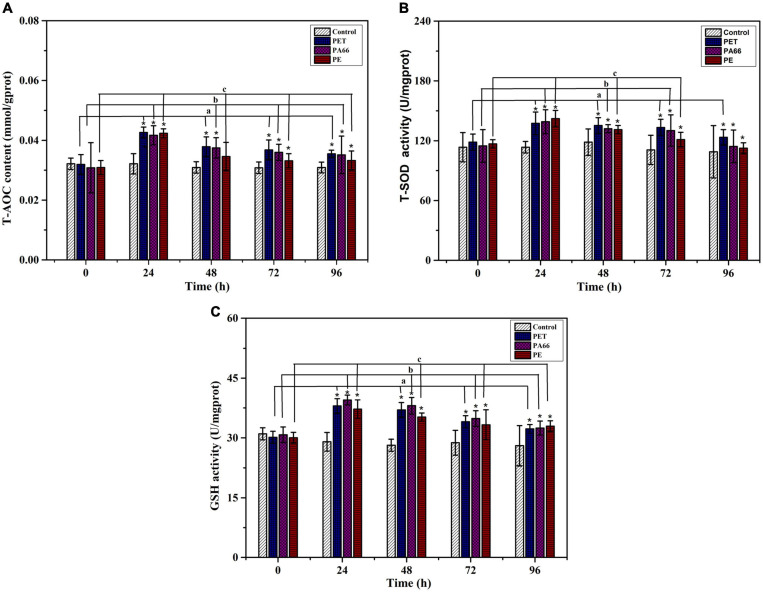
Changes in the anti-oxidative enzymes of corals after 96 h exposure to three kinds of microplastics. **(A)** Total antioxidant capacity (T-AOC), **(B)** Total Superoxide Dismutase (T-SOD), and **(C)** Glutathione (GSH) in the coral *Acropora pruinosa* after exposure to different MPs (PET, PA66, and PE 50 mg/L, respectively) in the experiment. Histogram data represent mean and error bars represent standard deviations (*N* > 3). Asterisk (*) represents a significant difference from normal (*p < 0.05).

Figures 4A–C show the anti-oxidative ability of coral tissue. As depicted in Figure 4A, the content of T-AOC (*p* < 0.05) was 4.26 × 10^–2^, 4.17 × 10^–2^, 4.23 × 10^–2^ mmol/mg prot after 24 h exposure in PET, PA66, and PE. Though it was gradually reduced after 48 h and 96 h, it was still higher than the control group. There was a significant increase in T-SOD activity after exposure 24 h (137.44 U/mg prot, 137.07 U/mg prot, 142.10 U/mg prot, *p* < 0.05) in PET, PA66, and PE groups. While it was decreasing after 96 h treatment (Figure 4B). Comparing with the control group, the GSH activities (*p* < 0.05) were higher in MPs groups after 24 h exposure. After 96 h, the GSH activities decreased (32.23 U/mg prot, 32.43 U/mg prot, 32.92 U/mg prot, *p* < 0.05) in MPs exposure groups (Figure 4C). In summary, the levels of T-SOD, T-AOC, and GSH increased after MPs exposure, indicating that MPs could induce coral defense against oxidative stress, which depended on the type of MPs.

#### The Effect of MPs on Alkaline Phosphate and Nitric Oxide

**FIGURE 5:**
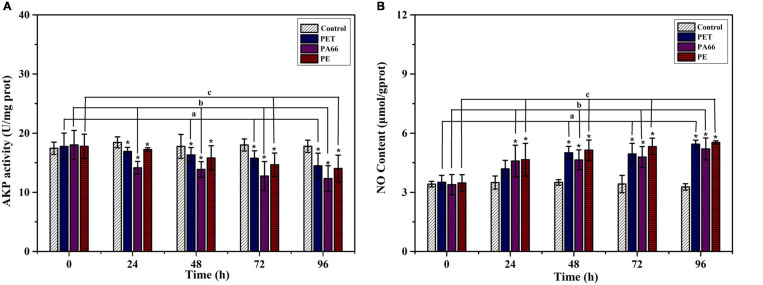
Immune ability analysis. AKP activity **(A)** and NO content **(B)** in the coral exposed to different MPs (PET, PA66, and PE 50 mg/L, respectively) for 0, 24, 48, 72, and 96 h. All data are presented as mean ± standard error (*N* > 3). Asterisk (*) represent a significant difference from normal (*p < 0.05).

As shown in Figure 5A, the AKP level showed an inhibiting trend throughout the experimental period (1.44–4.29 U/mg prot, *p* < 0.05) in all MPs exposure groups. At 96 h, the AKP activities (*p* < 0.05) were significantly decreased in MPs exposure corals when compared with the corals in the control group. On the contrary, the NO content in the MPs group sharply increased at 96 h (0.69–2.26 μmol/g prot, Figure 5B), and was higher (*p* < 0.05) than the control groups. It was ascending for NO content in all MPs exposure groups, indicating that the coral immune system may be sensitive to MPs.

#### The Effect of MPs on Glycolysis Enzymes (LDH) and Phosphate Pentoses Pathway (G6PDH)

**FIGURE 6:**
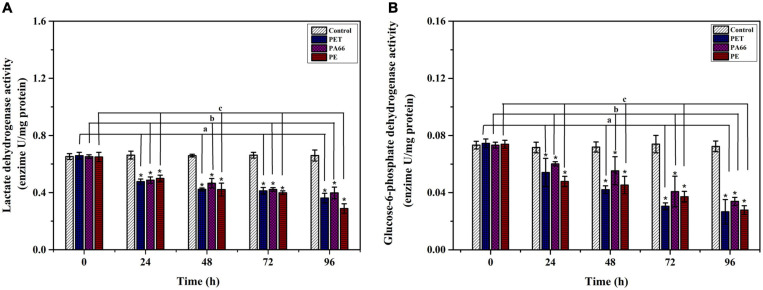
Energy metabolism enzyme analysis. LDH activity **(A)** and G6PDH activity **(B)** in the coral *Acropora pruinosa* after different MPs (PET, PA66, and PE 50 mg/L, respectively) exposure experiment. Histogram data represent means and error bars represent standard deviations (*N* > 3). Asterisk (*) represent a significant difference from normal (*p < 0.05).

An inhibiting effect was observed on LDH activity (*p* < 0.05) after exposure to MPs (Figure 6A) and decreased compared with the one in control coral at 24 h (0.19–0.22 U/mg prot, *p* < 0.05). The activity of LDH presented significantly lower values (*p* < 0.05) in MPs exposure groups at 96 h compared with the control group. As shown in Figure 6B, the G6DPH content (*p* < 0.05) decreased in MPs exposure corals compared with the corals in the control group at 24 h. The G6DPH content was sharply reduced (*p* < 0.05) in PET, PA66 and the PE group (0.01–0.04 U/mg port) at 96 h. This indicated that coral glycometabolism was influenced by the type of MPs.

## Discussion

The presence of MPs significantly affected the physiology of corals depending on the types of MPs (Hidalgo-Ruz et al., 2012; Hankins et al., 2018). In this study, we observed that the contents of MPs on the surface of the coral skeleton were significantly different from those of the control groups. Our study also discovered MPs in coral tissues, which aligns with other studies indicating that corals may ingest MPs (Allen et al., 2017; Tang et al., 2018; Reichert et al., 2019). Even though coral calcification depends largely on photosynthesis from the endosymbiont (Porter et al., 1989), the corals still supply carbon sources through predation (Grottoli et al., 2006; Anthony et al., 2009). MPs are ingested as food because they are not easy recognized by zooplankton or corals (Hankins et al., 2018). The large specific surface area and high hydrophobicity of MPs may increase the surface free energy of polar plastics and improve lipophilicity. Therefore, MPs can be adsorbed on the surface of corals (Ainsworth et al., 2007; Hidalgo-Ruz et al., 2012). MPs tend to accumulate on the surface of the coral skeleton, causing frictional damage and pathogen invasion (Chen et al., 2017; Reichert et al., 2018). They can also be ingested by the coral, leading to coral oxidative stress response or toxic effects (Chen et al., 2017; Tang et al., 2018). The toxic effect of MPs was determined by measuring the biochemical indicators of coral and further exploration of the relationship between the contents of MPs and physiological activities.

Exposure to MPs caused a stress response in the coral that further impaired its function. Firstly, the heterotrophic feeding may be inhibited due to excessive ingestion of MPs. Several studies have reported that the normal food supply of corals was hindered by MPs. The coral is unable to obtain nutrients owing to the continuous ingestion and excretion of MPs (Chapron et al., 2018; Reichert et al., 2019). When food sources are limited, the endosymbionts play a key role in the energy supply. The endosymbionts provide hosts with colors and energy because they can absorb light. They can also convert light energy into chemical energy through chlorophyll (Besseling et al., 2014; Pierson et al., 2017), meaning that endosymbionts play an important role in the growth and breeding of corals (Mcleod, 1957; Pierson et al., 2017). In the present study, the density of endosymbionts in coral decreased and maintained a stable density, which revealed that the corals could recover symbiotic balance in a short time. This is consistent with previous reports (Denis et al., 2013; Reichert et al., 2018; Corona et al., 2020). As expected, the chlorophyll content was reduced in coral symbiosis. The exchange of light energy was reduced due to the attachment of MPs on the surface of the coral skeleton. MPs can easily absorb the toxic metabolites of microorganisms that inhibit the activity of key photosystem II in coral (Hidalgo-Ruz et al., 2012; Mendrik et al., 2020). On balance, these stressors lead to the stress response of corals and their symbionts (Enriquez et al., 2005; Muller-Parker et al., 2015). The content of chlorophyll was closely related to the MPs on the surface of the coral skeleton. Furthermore, the toxicity of MPs increased and the symbiotic algae were reduced with time (Downs et al., 2002). Under different stressors, the chlorophyll content of scleractinian coral decreased temporarily, results that are confirmed by previous studies (Rocha et al., 2015; Long et al., 2017; Zhang et al., 2017; Lanctot et al., 2020). Lei believed that the change of chlorophyll content was one of the stress response indicators and that it reflected the density and photosynthetic capacity of the endosymbiont in corals (Lei et al., 2009).

Moreover, a disturbance of the symbiotic alga-host relationship could be caused by ROS from MPs stress (Okubo et al., 2018). In the antioxidant enzyme system of marine organisms, T-AOC, T-SOD, and GSH are important active enzymes that synergistically reduce the production of free radicals under negative stress (Levy et al., 2006; Sorianosantiago et al., 2013). The activities of T-SOD and T-AOC were up-regulated after MPs exposure at 24 h, meaning that MPs can induce ROS production and it enhances the anti-oxidative system of coral. The adverse effects of MPs, causing oxidative stress on scleractinian coral, have also been reported in previous research (Sorianosantiago et al., 2013; Tang et al., 2018; Liao et al., 2021). In the present study, there was the same tendency of oxidative stress caused by the three kinds of MPs. In general, the T-AOC content, T-SOD, and GSH activity showed the variation tendency of rising (Tang et al., 2018; Liao et al., 2021). After 96 h of exposure, the coral acclimation to MPs stress can occur through the production of antioxidant enzymes. The antioxidant system of corals (inactive damaging oxygen radicals) can scavenge denatured cellular proteins to reduce the harm of active free radicals (Hofmann and Todgham, 2010; Weis, 2010). The corals can resist external adverse factors by enhancing antioxidants or self-cleaning (Denis et al., 2013; Rocha et al., 2015). When a mass of MPs are ingested by corals, they cause damage to the coral tissues because they accumulate in the gastrointestinal tract and liposomes of corals (Hall et al., 2015). Previous reports have shown that oxidative damage of coral symbiosis increased in the presence of MPs (Jeong et al., 2016; Tang et al., 2018). Tang outlines that exposure to MPs may regulate the JNK and ERK signaling pathways and that it inhibits the phosphorylation process in corals (Tang et al., 2018). It is worth mentioning that these effects directly weaken coral’s ability to detoxify. GSH is a small molecule peptide composed of three amino acids, and it is a special substance for detoxification (Krueger et al., 2014; Nicosia et al., 2014). GSH activity increased under MPs exposure at 24 h. Finally, it was decreased in MPs exposure groups at 96 h. This indicated that short-term MPs exposure could regulate the detoxification system in *Acropora* sp. Under MPs stress, the detoxification system was disturbed, which accelerated the collapse of the host-symbiotic algae relationship (Tang et al., 2018). In summary, MPs could activate the *Acropora* sp. antidotal system in a short time.

The immune system is the host defense system, and AKP is an essential enzyme in corals. It participates in the identification and clearance of pathogenic organisms or materials (Palmer et al., 2011). In scleractinian corals, the immune function of AKP has been confirmed (Palmer et al., 2011; Godinot et al., 2013). Oxidative stress is expected to aggravate the negative immunity effects of MPs exposure on corals (Godinot et al., 2013; Tang et al., 2018). Our results suggested that there was a significant correlation between MPs and AKP in *Acropora* sp. The AKP activities in all MPs exposure groups showed a decreasing trend in the experiment. AKP activity decreased significantly, which suggested that MPs-induced stress may damage the immune system of *Acropora* sp. by regulating the oxidative stress signal pathway. Studies have revealed that the oxidative stress caused by MPs exposure can lead to the occurrence of immunosuppression in corals (Tang et al., 2018). Moreover, some similar results have been reported in other species (Detree and Gallardo-Escarate, 2018; Liao et al., 2021). An imbalance in immunity capacities was induced by MPs, which could disturb pathways or genes of the immune system in coral. Tang’s results showed that MPs impacted the immune system in corals by regulating the MAPK signal pathways (Tang et al., 2018). However, the mechanism of immune system suppression still requires further study and research. Besides, NO is the product of inflammatory molecules in corals, which have a significant effect on coral bleaching (Perez and Weis, 2006). When exposed to MPs, the NO content was increased in coral, meaning that the pressure on the immune system in corals increased. The rapid increase of NO in the host may adversely affect the symbiosis of coral-endosymbiont (Perez and Weis, 2006; da Silva Fonseca et al., 2019). The role of NO not only involves host apoptotic-like cell death but also receiving and transmitting information. It can regulate the activity of the host-endosymbiont cells (Hawkins and Davy, 2012; Hawkins et al., 2014; da Silva Fonseca et al., 2019). These results indicate that an immunosuppressive effect could be caused by MPs in the coral host. In addition, the synthesis NO from the host was related to the health of the coral-endosymbiont.

The LDH and G6PDH in coral *Acropora* sp. were investigated to understand the effects of MPs exposure on the energy metabolism of corals. In the presence of MPs, the content of glycolytic enzyme (LDH) was reduced, indicating that the inhibition of LDH activity can compromise the coral energy metabolism. In turn, it reduced the aerobic metabolism because organisms failed to initiate physiological adjustment that leads to severe anaerobic metabolism. The exposure of MPs had an overall inhibitory effect on the enzyme activities related to energy metabolism in corals (Teuten et al., 2009; Andrady, 2011). Therefore, the change of LDH activity directly affected the corals’ energy metabolism (Hosseini et al., 2014; da Silva Fonseca et al., 2019). In addition, G6PDH is responsible for the production of ribose units necessary for nucleotide synthesis that contributes to antioxidant system, lipid synthesis and, bioconversion (Carvalho and Fernandes, 2008; Nelson et al., 2008). After 24 h exposure to MPs, the activity of G6PDH was suppressed, resulting in metabolic and oxidative damages of coral. Besides, a consistent decrease of enzymatic activity was observed after 96 h exposure to MPs. The results from enzymatic activities showed that exposure to stressors induce a state of energy limitation in the scleractinian coral *Acropora* sp. An insufficient energy supply can accelerate the collapse of the symbiotic system of corals.

The results of this study indicate that short-term exposure to high concentrations of MPs could induce the stress response of scleractinian coral *Acropora* sp. as well as inhibit the activity system of major enzymes in energy metabolism. Based on our results, it is clear that short-term exposure to concentrations of MPs is the potential to cause metabolic dysfunction between *Acropora* sp. and its algal symbionts. However, long-term exposure to lower concentrations *in situ* still needs to be studied.

## Conclusion

In conclusion, the present study revealed that there were correlations between MPs exposure and physiological parameters in corals *Acropora* sp. The number of MPs ingested by corals was significantly different among PET, PA66, and PE. MPs exposure disrupted the balance between symbiosis and corals by influencing the density of endosymbiont and chlorophyll. The antioxidant enzyme T-AOC content, T-SOD activity, and GSH activity were maintained at higher levels, which suggested that MPs caused the breakdown of the oxidation-reduction enzyme balance in the coral and endosymbiont symbiosis. The AKP enzyme was inhibited to various degrees by MPs. The content of NO in whole MPs exposure groups increased in the whole exposure experiment, which revealed that the immune functions suffered disruption to some extent. LDH activity was significantly down-regulated, which indicated that the energy metabolism and homeostasis of corals were disturbed. The variation of G6PDH activity showed that the coral *Acropora* sp. phosphate pentoses pathway was destroyed. The results showed the MPs ingested by corals would lead to the destruction of oxidative stress, immune suppression, and energy metabolism pathways.

## Data Availability Statement

The original contributions presented in the study are included in the article/Supplementary Material, further inquiries can be directed to the corresponding author/s.

## Author Contributions

BX: conceptualization and funding acquisition. DL: conceptualization, writing the original draft, and writing, review, and editing the manuscript. CL: supervision, project administration, and funding acquisition. BL: investigation and formal analysis. HZ: data curation. XY: methodology. YX: software, supervision, and validation. ZX: investigation and formal analysis. All authors contributed to the article and approved the submitted version.

## Conflict of Interest

The authors declare that the research was conducted in the absence of any commercial or financial relationships that could be construed as a potential conflict of interest.

## Footnotes

1 https://www.aliexpress.com/i/4000118987595.html

## Supplementary Material

Supplementary Figure 1 The microscope images and size distribution of three types of MPs (PET, PA66, PE).

Supplementary Figure 2 The coral reef field locations (22°33′50.78″−22°40′38.18″N, 114°30′35.62″−114°33′26.90″E) and sample (*Acropora* sp.).
