# Supplementary material for: Effects of Microplastics Exposure on the Acropora sp. Antioxidant, Immunization and Energy Metabolism Enzyme Activities
Source: Front Microbiol. 2021 Jun 4;12:666100. doi: 10.3389/fmicb.2021.666100 (PMC8213336; doi:10.3389/fmicb.2021.666100)
Supplement: Supplementary Figure 1 — The microscope images and size distribution of three types of MPs (PET, PA66, PE). [file Data_Sheet_1.docx]

**Effects of microplastics exposure on the *Acropora* sp. antioxidant, immunization and energy metabolism enzyme activities**

Baohua Xiao^a,1^, Dongdong Li^a,1^, Baolin Liao^a^, Huina Zheng^a^, Xiaodong Yang^a^, Yongqi Xie^a^,

Ziqiang Xie^a^, Chengyong Li^a, b^[[1]](#footnote-1)^*^

a. Shen Zhen Institute of Guangdong Ocean University, Shenzhen 518120, Guangdong, People’s Republic of China.

b. School of Chemistry and Environment, School of Food Science and Technology, College of Coastal Agricultural Sciences, Guangdong Ocean University, Southern Marine Science and Engineering Guangdong Laboratory, Zhanjiang 524088, Guangdong, People’s Republic of China.

***
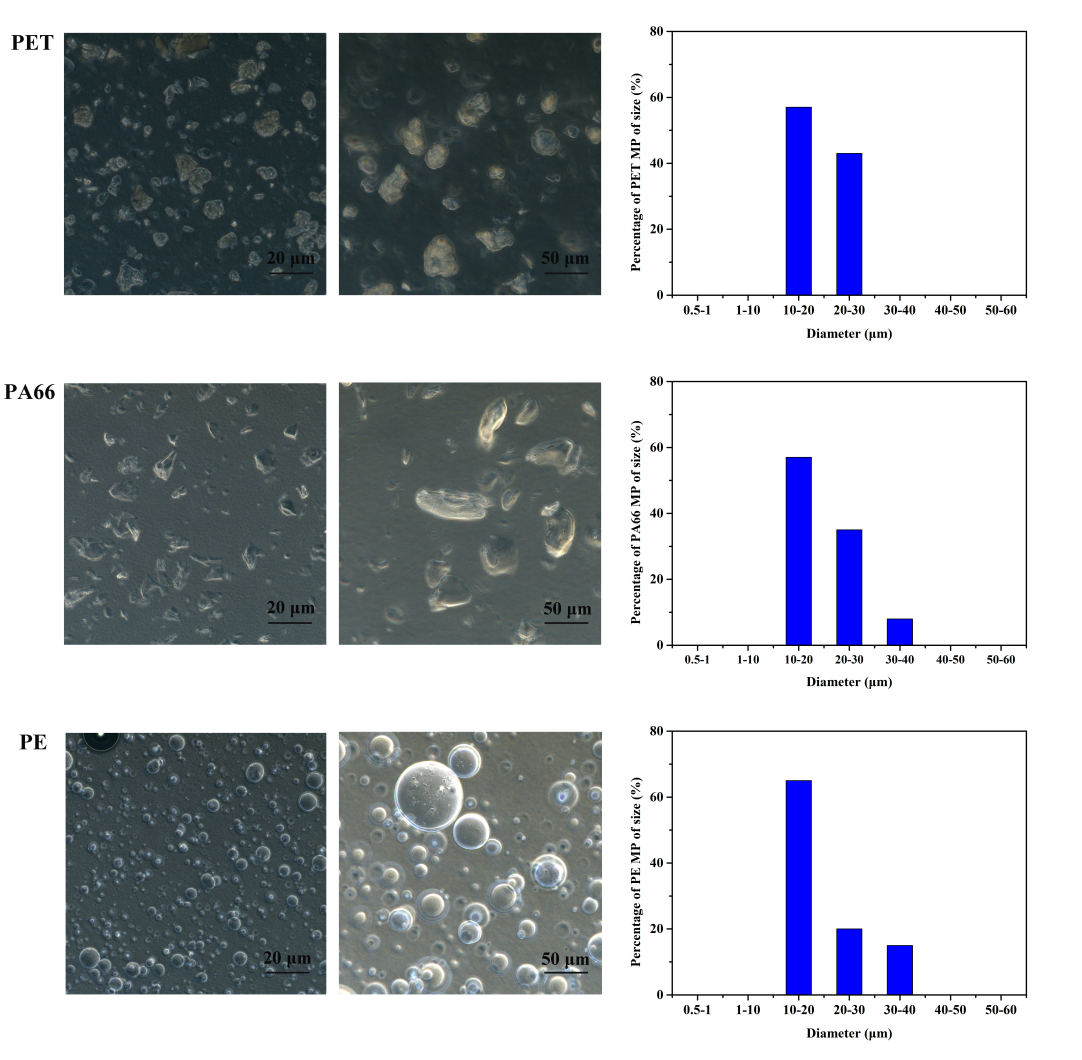
***

Fig. S1. The microscope images and size distribution of three types of MPs (PET, PA66, PE).

***
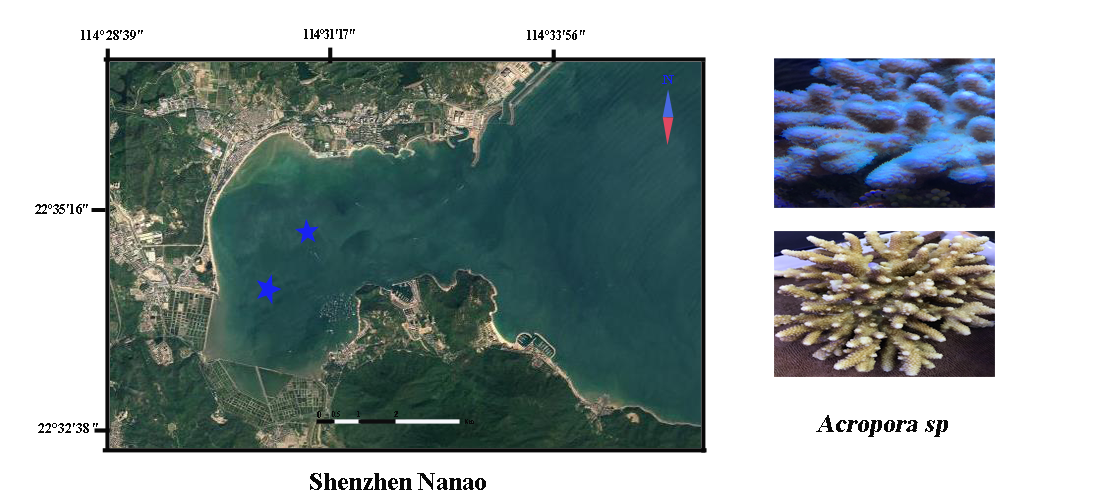
***

Fig. S2. The coral reef field locations (22°33′50.78″-22°40′38.18″N, 114°30′35.62″-114°33′26.90″E) and sample (*Acropora* sp).

1. ^*^Corresponding author: [cyli@gdou.edu.cn](mailto:cyli@gdou.edu.cn) (Chengyong Li)

   1 These authors contributed equally to this work. [↑](#footnote-ref-1)
